# Exploring the Spatiotemporal Evolution and Sustainable Driving Factors of Information Flow Network: A Public Search Attention Perspective

**DOI:** 10.3390/ijerph19010489

**Published:** 2022-01-02

**Authors:** Fei Ma, Yujie Zhu, Kum Fai Yuen, Qipeng Sun, Haonan He, Xiaobo Xu, Zhen Shang, Yan Xu

**Affiliations:** 1 School of Economics and Management, Chang’an University, Xi’an 710064, China; mafeixa@chd.edu.cn (F.M.); sunqip2003@163.com (Q.S.); haonanhe@chd.edu.cn (H.H.); shangzhen@chd.edu.cn (Z.S.); xuxiaoyan0125@126.com (Y.X.); 2 School of Civil and Environmental Engineering, Nanyang Technological University, Singapore 639798, Singapore; kumfai.yuen@ntu.edu.sg; 3 International Business School, Xi’an Jiaotong-Liverpool University, Suzhou 215123, China; Xiaobo.Xu@xjtlu.edu.cn

**Keywords:** information flow, public search attention, intercity network, driving factors, urban sustainable development

## Abstract

The promotion of information flow reinforces the interactive cooperation and evolutionary process among cities. In the information age, public online search is a typical behavior of Internet society, which is the key to information flow generation and agglomeration. In this study, we attempt to explore the evolutionary characteristics of intercity networks driven by public online social behavior in the information age and construct an information flow network (IFN) from the perspective of public search attention. We also explore the evolution of the IFN in terms of the whole network, node hierarchy, and subgroup aggregation. Meanwhile, we also discuss the impact of the sustainable driving factors on the IFN. Finally, an empirical study was conducted in Guanzhong Plain Urban Agglomeration (GPUA). Our results show that: (1) the information flow in GPUA fluctuating upward in the early study period and gradually decreasing in the later study period. However, the agglomeration degree of information flow in the urban agglomeration continues to increase. (2) The hierarchical structure of urban nodes in GPUA presents a trend of “high in the middle and low on both sides”, and the formation of subgroups is closely related to geographic location. (3) The driving factors all impacting the IFN include public ecology, resource investment, information infrastructure, and economic foundation. This study provides theoretical and practical support for exploring the intercity network and promotes the sustainable urban development.

## 1. Introduction

Information technology and the Internet have significantly reshaped people’s social production activities [1]. The flow, agglomeration and dependence of intercity human, logistics, information, and capital flows and other factor flows are enhanced, promoting the networking of urban spatial organization [2]. The Internet has gradually emerged as the main tool for information production and dissemination, which has led to a stream of information flowing without any geographic spatial constraints [3]. Under this circumstance, the information flow promotes the networked evolution of the spatial structure among cities more rapidly [4]. Therefore, analyzing the spatiotemporal evolution of intercity networks from the perspective of information flow is the key to uncovering intercity interaction relations, coordinating urban planning and functional positioning, and achieving sustainable urban development in the information age.

Meanwhile, as a typical Internet query tool, the search engine with the characteristics of being instant, rapid, and convenient, has become an important platform for the public to query and obtain information [5]. Statistics indicate that China had 790 million users of Internet search engines in 2020, with 7 billion average daily search requests [6]. It shows that the public demand for information acquisition is increasing rapidly, and people tend to use search engines to query and obtain various information according to their attention and needs. The Internet search with the characteristics of real-time and high-frequency not only generates massive search information but also reflects the attention of the public search [7,8]. Furthermore, Internet search depicts the personal behavior of netizens in the online society, which becomes one of the important sources of Internet information flow. Therefore, we can reveal the information flow among cities through the information generated by the public search attention [9,10], which further reflects the evolution of the intercity network.

At present, the indexes commonly used to characterize information flow in related research mainly focus on Baidu Index and Google Trend. Table 1 shows the characteristics of the Baidu Index and Google Trend [11,12,13].

Based on the factors listed in Table 1, we decided to use Baidu Index (BI) as the representative of public search attention to reflect the characteristics of information flow. The reasons include: (1) the BI being very easy to obtain and query for its accurate data and obvious performance [14]. Meanwhile, the BI can intuitively quantify the search attention of each keyword and provide good data support for research [15]. (2) The BI is widely used in China, generating massive data. The massive data support reflects Internet users’ views and attention on certain topics more accurately, identifying the public online social behavior effectively [16]. (3) Using BI is of high research value for a deeper understanding of the impact of public social behavior changes on the intercity network in the information age.

This study focuses on the impact of public social behavior on intercity networks in the information age. We also attempt to characterize information flow from the public search attention perspective to explore the characteristics of evolution and hierarchical structure of intercity network. Based on this, we proposed a research framework for the spatiotemporal evolution and driving factors of intercity network from the information flow perspective. Firstly, we used BI to represent information flow and construct an information flow network from the perspective of public search attention. Secondly, structural holes, dominance flow analysis, and cohesive subgroups were used to analyze the evolution of the information flow network from the perspective of whole network, node hierarchy, and subgroup aggregation. Finally, we used quadratic assignment procedure (QAP) analysis to explore the influence of various driving factors on the sustainable development of the information flow network. The research results provide a new direction for future urban interactive cooperation and sustainable development in the information age.

## 2. Literature Review

(1) The intercity network from the perspective of “flow” space

Exploring the urban spatial organization structure and network characteristics is a research hotspot in the field of urban geography [17,18]. The early intercity network was mainly researched using static geographic spatial attributes such as urban hierarchical structure and location characteristics, which ignored the impacts of factor flows on the intercity network [19]. Castells first proposed the concept of “flow space”, which clarified that urban space is a spatial organization structure composed of flow elements such as people flow, logistics flow, capital flow, information flow, and technology flow [20]. After this point was put forward, many scholars analyzed the profound impact of factor flows on urban spatial organization, social environment changes, and residents’ life behavior from the perspectives of people flow, traffic flow, and logistics [21,22,23,24]. However, the existing research mainly focuses on the flow of material carrier elements (people flow, product flow, traffic flow, etc.). There is a relatively lack of research on exploring the characteristics of information flow among cities. With the advent of the information age, information technology has strengthened the interconnection among regions, which in turn affects the regional hierarchy and connection pattern [25]. Exploring the spatial connection among cities from the perspective of information flow has become a research hotspot in urban geography. Some scholars began to analyze the spatial interaction structure under the influence of information technology from information flow perspective, emphasizing that information technology intensified the interaction of urban space. For example, Dadashpoor et al. explored the spatial effects of information and communication technology on intercity elements and functions [26]. Huang et al. measured the diffusion of cross-country information traffic on the Internet based on weekly moving data across countries [27]. However, these scholars are mainly focusing on the impact of information technology on the intercity networks, which did not further load the information flow into the intercity network to discuss the evolution of the intercity network and the interactive characteristics among cities [28].

(2) New exploration under the public search attention

With the support of abundant big data, choosing appropriate indicators to quantify information flow and explore intercity networks is the key to solve our research problem. The popularity of the Internet has produced a wealth of behavioral data, which provides important data support for the quantitative measurement of intercity network [29]. For example, Weibo data are used to explore the spatial network strength characteristics of urban agglomerations [30]; Tencent location data are used to analyze complex intercity network characteristics under migrate flow [31]. However, Weibo data and Tencent location data have a data processing problem in accurately quantifying information flow. Meanwhile, with the widespread use of search engines on the Internet, an Internet search index that records the search behavior of netizens came into being, which is widely used in the research of economic geography, human geography, and other fields [32,33]. However, most of the current research applied the Internet search index is meant to monitor the public opinion of netizens and dig out hot topics of public concern [34,35]. Few scholars have noticed the underlying mechanism of Internet search behavior and the urban spatial evolution in the information age. Actually, as a typical social behavior in the information age, the Internet search has made a huge contribution to the generation of information flow. The search index reveals the public search attention and interest in a certain topic, which is related to the public actual social behavior and further affects the urban spatial organization structure [36]. Therefore, exploring the interactive in the spatial organization structure among cities according to the public search attention has become a new research direction [37,38].

(3) Driving factors of the intercity network

With scholars’ in-depth discussion on the evolution and interaction of the intercity network, the factors that drive changes in the urban spatial structure have also become important research directions. The intercity network is affected by a variety of influencing factors, and identifying the effects and mechanisms of influencing factors plays an important role in effectively improving the coordinated development of cities. Wu explored the spillover effects of the intercity network from four aspects: urban size, economic foundation, major events, and geographic space [39], while Wang analyzed the influencing factors from the dimensions of economic scale, population scale, infrastructure, urban function positioning, urban event effects, and historical culture [40]. However, each study does not have a unified standard when constructing the driving factors of the intercity network, which will be adjusted rationally and scientifically according to the focus of the research content. In addition, economic foundation and infrastructure are the important lifeline and source of vitality of the city, playing an important role in the interaction of the intercity network space structure [41]. Therefore, most scholars choose economic foundation and infrastructure as basic factors to measure the impact on intercity network. With the advancement of the construction of national ecological civilization, urban sustainable development has become an important force for urban renewal and stability maintenance [42,43]. A few scholars began to further explore the relationship between urban sustainable development and urban spatial structure from the perspectives of air pollution, energy reduction, green ecology, and coupling and coordination [44,45,46]. However, previous research discussed the factors that influence the sustainable development of intercity networks from a single-dimensional driving factor. These studies are generally lacking a comprehensive exploration of the key factors driving urban sustainable development. Therefore, further explaining the factors affecting the sustainable development of the intercity network is a new requirement for improving urban vitality and urban ecological health.

In summary, existing research of intercity networks mainly discusses the formation and evolution of intercity networks from the perspective of physical carrier element flow. However, few studies have explored the spatiotemporal evolution and interactive connection of intercity network based on virtual information flow. Furthermore, people are important participants in the intercity networks that control the flow of elements. Especially in the information age, people’s social behavior has undergone major changes. However, the existing research seldom explores the relationship between people’s social behavior and intercity network structure. Therefore, it is necessary to analyze the effect of typical people’s social behavior (i.e., Internet search behavior) on the Internet on the intercity network. Meanwhile, sustainable development is an important topic for urban renewal and maintaining vitality, but there is limited research that explores the relationship between sustainable development and the evolution of the intercity network from the multiple dimensions. Therefore, to fill the above gaps, we selected BI (a typical Internet search behavior indicator) as the data support and built an information flow network based on the information flow from the perspective of public search attention, highlighting the important role of search behavior in the Internet social behavior. Furthermore, we also explored the influencing factors of the intercity network for the goal of urban sustainable development.

## 3. Research Methods and Data Sources

### 3.1. Research Methods

**Figure 1:**
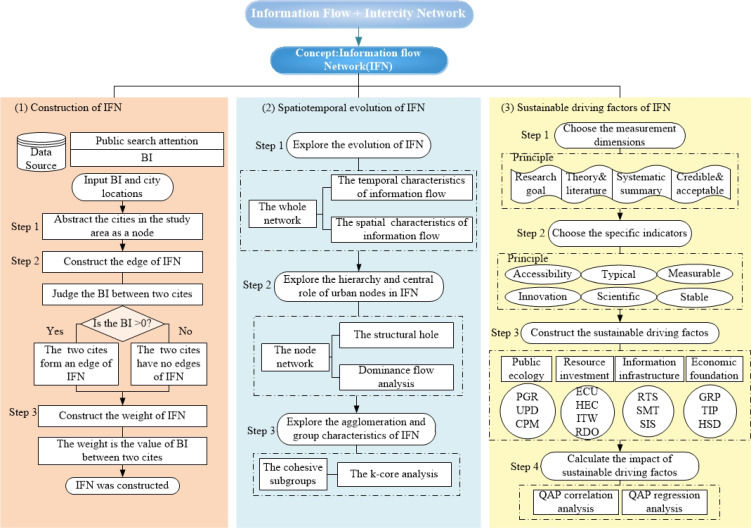
The research framework.

We constructed a feasible research framework to explain the evolution of intercity network based on information flow and the impacts of urban sustainable driving factors from the perspective of public search attention. Particularly, the concept of information flow network (IFN) was proposed in this research; meanwhile, BI, an index that quantifies public search attention reflecting information flow, was loaded into the IFN as data support. Furthermore, we measured the spatiotemporal evolution and urban role of the IFN from three aspects: the whole network, the nodes hierarchy, and the aggregation of subgroups. Finally, to explore the impact of driving factors for urban sustainable development, a correlation analysis and regression analysis were carried out between the sustainable driving factors and the IFN. The research framework proposed in this paper is shown in Figure 1.

#### 3.1.1. The Model Construction of IFN

The IFN is a complex network composed of “nodes” and “edges”, which are also a collection of interrelationships between each city’s information flows. The “nodes” denote the cities, and the “edges” denote the spatial association of the information flow among cities [47,48]. Therefore, the IFN is described as a tuple expression: *IFN = <V*, *E>*. The *V = {v*_1_, *v*_2_,*…*, *v_n_}* denotes the node set of IFN; the *E = {e_ij_ = (v_i_*, *v_j_)| v_i_*, *v_j_* ∈ *V}* denotes the edge set of IFN. The detailed construction process of IFN is as follows:

Step 1: abstract each city into a network node;

Step 2: construct the edges of IFN based on the threshold of BI. To explore the characteristics of the whole network, we set the threshold to 0. Meanwhile the “edges” are represented by Equation (1).

(1) $$e_{\mathrm{ij}}=\left\{ \begin{array}{l} 1,\mathrm{BI}>0\\ 0,\mathrm{BI}\leq 0 \end{array}\right.$$

Step 3: define the weight of “edge”. Furthermore, we defined the matrix as the edge weight matrix of IFN; the *w_ij_* is the weight of the edge *e_ij_*. The *w_ij_* is measured by the BI, representing the information flow of *e_ij_*. The matrix *W* is presented by Equation (2):

(2) $$W=\left[\begin{array}{cccc} 0 & w_{12} & \cdots & w_{1n}\\ w_{21} & 0 & \cdots & w_{2n}\\ \vdots & \vdots & \ddots & \vdots \\ w_{n1} & w_{n2} & \cdots & 0 \end{array}\right]$$

where *n* represents the number of nodes in the network.

**Figure 2:**
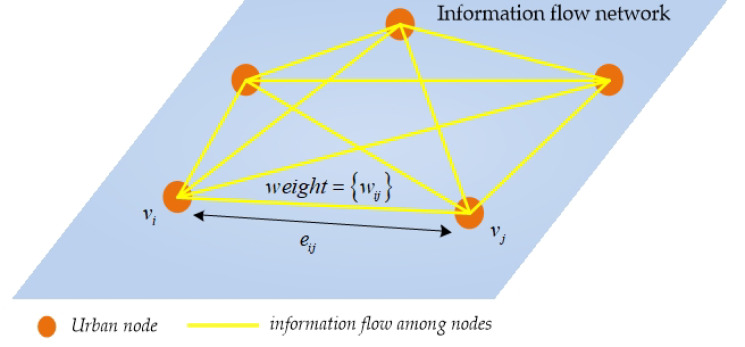
The structure of information flow network (IFN).

Based on the above steps, Figure 2 shows the structure of IFN.

One point worth emphasizing is that the information flow constructed by BI between two cities is bilateral. However, in this study, we considered the IFN as an undirected network. To eliminate the direction of information flow, we calculated the *w_ij_* by the sum of the information flow for city *i* and *j*. The *w_ij_* is presented by Equation (3):

(3) $$w_{ij}=r_{ij}+r_{ji}$$

where *r_ij_* is the information flow of city *i* to city *j*; *r_ji_* is the information flow of city *j* to *i*. Then, the whole information flow of node *i* was calculated by the sum of the edge weight of *e_ij_*. The whole information flow of node *i* is presented by Equation (4):

(4) $$C_{i}=\sum_{j=1}^{n}w_{ij}$$

where *C_i_* is the whole information flow of city *i*. The whole information flow of IFN is represented by the sum of *C_i_* using the Equation (5):

(5) $$C_{total}=\sum_{i=1}^{n}C_{i}$$

Finally, we also measured the dispersion of information flow by the coefficient of variation. The coefficient of variation (*C_v_*) is a statistical indicator used to measure the degree of data dispersion. The *C_v_* measures the differences in the state of data over different time segments and across different regions. The larger the value of *C_v_*, the higher the degree of dispersion of information flow. We used *C_v_* to indicate the coefficient of variation of information flow in Equation (6):

(6) $${\text{C}}_{\text{v}}=\text{SD}/\text{MN}$$

where *SD* is the standard deviation of information flow and *MN* is its average value.

#### 3.1.2. The Structural Holes

The structural holes theory is one of the common theories used in network structure analysis [49,50]. The theory of structural holes suggests that if there are no direct connections between node A and node B, then there is a structural hole between A and B. Meanwhile, if a node C, which connects node A and B, serves as an intermediary, then node C has occupied the structural hole with more resources and connections. Therefore, an urban node with more structural holes indicates that it has gained more resources and connections and is in a dominant position. According to the research results of previous scholars, the typical measurement indicators of structural holes are “indicators of Burt” and “centrality indicators”. Burt proposed that the calculation of structural holes needed to consider four relevant indicators: effective size, efficiency, constraint, and hierarchy [51]. Centrality, as an important indicator to measure the core degree of nodes in the network structure, reflects the core position of the node in the structural holes [52]. Table 2 shows the specific meaning of typical structural holes indicators.

**Figure 3:**
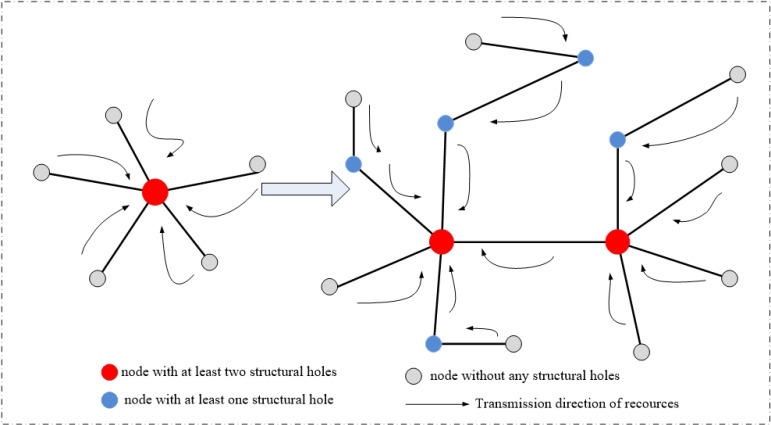
The explanation of structural holes theory.

According to Table 2, “effective size”, “efficiency”, and “constraint” measure the three aspects of the structural holes including the degree of occupied resources, the conversion efficiency, and the degree of restriction, respectively. “Hierarchy” refers to the agglomeration of constraints on nodes to indirectly reflect the core degree of nodes, with the same role as “constraint”. “Centrality” is a measurement indicator of the core degree of the connection among nodes, which more directly reflects the hierarchy of nodes. Therefore, considering the principle of comprehensiveness and optimization, we focused on four perspectives of structural holes: effective size, efficiency, constraint, and centrality. Figure 3 explains the structural holes theory.

(1) The effective size (*ES_i_*): The *ES_i_* represents nonredundant factors in the network. The node’s effective size is the node’s individual network size (i.e., the number of nodes related to the ego node) minus the redundancy of the network. The *ES_i_* was calculated using Equation (7):

(7) $$ES_{i}=\sum_{j}\left(1-\sum_{q}p_{iq}m_{jq}\right),q\neq i,j$$

where *i* represents an ego node *i*, and *j* represents a node related to node *i*; the *q* is any node except *i* and *j.* Only the relationship between the nodes *i* and *j* is regarded as a valid relationship, while the relationship between other nodes is regarded as a redundant relationship. Therefore, the *p_iq_* represents the redundant relationship between node *i* and *q*. The *m_jq_* represents the redundant relationship between node *j* and *q*. The *p_iq_m_jq_* represents the redundancy between node *i* and *j.*

(2) The efficiency (*E_i_*): The *E_i_* is measured by the ratio of the node’s effective size to the node’s actual size in the individual network. The *E_i_* is calculated using Equation (8):

(8) $$E_{i}=ES_{i}/S_{i}$$

where *E_i_* represents the effective size of node *i*, and the *S_i_* represents the actual network size of node *i*.

(3) The constraints (*C_ij_*): The *C_ij_* measures the restriction of the node by other network nodes. The *C_ij_* is calculated using Equation (9):

(9) $$C_{ij}={\left(p_{ij}+\sum_{q}p_{iq}p_{qj}\right)}^{2}$$

where *i* represents an ego node *i*, and *j* represents a node related to node *i*; the *q* is any node except *i* and *j*. Only the *p_ij_* represents the valid relationship between node *i* and *j*. The *p_iq_* is the redundant relationship between node *i* and *q*. The *p_qj_* is the redundant relationship between node *q* and *j*.

(4) The degree centrality (*C_D_*(*n_i_*)): Centrality is a common indicator to measure the importance of urban nodes, which includes the degree centrality, closeness centrality, and betweenness centrality. In this study, we used degree centrality to measure the core degree and rank status of nodes in the network. The degree centrality was calculated using Equation (10):

(10) $$C_{D}(n_{i})=k(i)$$

where *C_D_*(*n_i_*) represents the degree centrality of node *i*; the *k(i)* represents the number of nodes that have a direct information flow with node *i* in the network.

#### 3.1.3. Dominance Flow Analysis

Dominance flow analysis is a relatively mature graph method for exploring the hierarchical structure of urban nodes, which has been widely used in the urban hierarchical structure. Dominance flow analysis supports that there are many “flows” in the network, with different scales and directions. Furthermore, Nystuen and Dacey proposed that each urban node will generate the largest-scale flow with other urban nodes, which is defined as the “dominant flow” in the network [53]. Dominance flow analysis extracts the main node hierarchy of the network by calculating the dominance flow of each node in the network. In this study, there are many kinds of “information flow” in IFN, and information flow generates from one city to another city. In the dominance flow analysis, we did not ignore the direction and scale of information flow. We defined the largest-scale information flow in the IFN as the “first dominant flow” and the second-scale information flow as the “second dominant flow”. For example, the information flow from node A to node B is the largest-scale comparing to the other nodes, which indicates that node B receives a “first dominant flow”. Furthermore, each urban node is divided into dominant nodes, subdominant nodes, and subordinate nodes according to the number of dominant flows received. The specific calculated process and affiliation of urban nodes are shown in Table 3 and Table 4 respectively.

#### 3.1.4. The Cohesive Subgroups Analysis

The “social solidarity” and “social cohesion” are core concepts in classical sociological research. Cohesive subgroups analysis is an important method for exploring the clustering phenomenon in social networks. To study the relationship of subgroups in the network structure, we analyzed the cohesive subgroups in the network. Cohesive subgroups are collections of nodes with specific agglomerated relationships, which are measured by the reachability, proximity, density, and degree centrality. In this study, we used the *k*-core algorithm that measured the degree centrality of each urban node to identify core members and cohesive subgroups with a specific *k* core degree. A *k*-core is defined as a subgroup of nodes with the same degree of centrality *k*, where *k* = 1,2,3...*n*. In the final sub-graph of the *k*-core, each node has at least the degree of *k*, and all nodes are connected to at least a number of *k* nodes in the subgraph. Therefore, we defined the set of urban nodes with the same degree of centrality *k* as cohesive subgroups; the urban nodes with the largest degree of centrality *k* are the core members.

#### 3.1.5. The *QAP* Analysis

##### The Model of QAP

We used the “relational” data (i.e., BI) to explore the intercity network because they are related to each other, unlike the attribute data. The IFN denotes the information flow among intercity, while the geographical proximity among cities may cause collinearity in the regression results. Furthermore, the QAP analysis can eliminate the effects of collinearity. The QAP is a quantitative method for measuring and analyzing relational data, which indicates the relationship between matrices. The QAP analysis conducts the correlation and regression analysis by comparing the similarity of matrix values with multiple transpositions, which also measures the standardized regression coefficients and significance among multiple matrices. Therefore, the QAP analysis is a widely used method in previous research. The model of QAP is shown as Equation (11):

(11) $${R=f(x}_{1}{,x}_{2}{,x}_{3},...{,x}_{n})=\partial_{1}x_{1}+\partial_{2}x_{2}+\partial_{3}x_{3}+...+\partial_{n}x_{n}+\varepsilon$$

where *R* represents the matrix of dependent variable matrix, and $x_{1},x_{2},x_{3},...,x_{n}$ are the matrices formed by each independent variable. The $\partial_{1},\partial_{2},...\partial_{n}$ represent influence coefficients of this model, and the ε represents the random coefficient.

##### The Construction of Sustainable Driving Factors

The sustainable development of intercity networks is important for optimizing urban planning. Economic foundation and infrastructure are the basic factors that affect the intercity network, while ecology and resources are key factors to measure urban sustainable development. Therefore, according to the four principles, research goals, theory and literature, systematic summary, and credible and available, the dimensions of the sustainable driving factors are constructed as: (1) public ecology, (2) resource investment, (3) information infrastructure, and (4) economic foundation. Furthermore, 13 specific measurement indicators are selected according to the principles of accessibility, typical, measurable, innovation, scientific, and stable.

(1) Public ecology. Public ecology is a critical factor affecting the sustainable development of cities. Measuring the state of urban public ecology is an important way to plan urban resources. Rapid population growth and population density as the typical indicators of measuring population size indirectly reflect the growth of urban resource consumption, which also affect the urban sustainable development. Transportation is an important tool for urban residents’ production activities, and transport generators also play an important role in affecting urban public ecology [54]. However, due to the wide range of transport generators, it is difficult to directly measure the impact of transport generators with a qualitative indicator. Therefore, we choose “annual mean concentration of PM2.5” as a comprehensive indicator to indirectly reflect the impact of urban transport generators on the ecology [55]. As an important indicator of air pollution, the annual mean concentration of PM2.5 can also reflect potential information such as urban traffic production, which also reveals the important role of transportation and industry in affecting public ecology. Therefore, we used three indicators to represent public ecology: population growth rate, population density, and annual mean concentration of PM2.5.
(2) Resource investment. Resource investments affect the clean production of cities, especially the input and consumption of energy. The consumption of power resources affects the sustainable development of cities. Investments in human resources and capital enhance the information flow. Therefore, we used four indicators to represent resource investments: energy consumption per unit of GDP, household electricity consumption, number of information technology workers, and internal outlays of R&D.
(3) Information infrastructure. Information infrastructure is an important factor that affects intercity information flow. In the information age, the provision of information infrastructure is important for the sustainable development of IFN. Therefore, we used three indicators to represent information infrastructure: revenue from telecommunication services, number of mobile telephone subscribers, and number of Internet services subscribers.
(4) Economic foundation. Economic development is the foundation for achieving sustainable development. Therefore, the economic foundation is an important factor for assessing urban sustainable development. The life quality of residents is one of the important aspects to measure the economic foundation. In this study, we used three indicators to represent the economic foundation: per capita GDP, tertiary industry as a percentage of GDP, and household saving deposits. Table 5 shows the sustainable driving factors considered for the information flow.

Based on the above theories, we constructed the following specific model:

(12) $$W=\partial_{1}\text{PGR}+\partial_{2}\text{UPD}+\partial_{3}\text{CPM}+\partial_{4}\text{ECU}+\partial_{5}\text{HEC}+\partial_{6}\text{ITW}+\partial_{7}\text{RDO}+\partial_{8}\text{RTS}+\partial_{9}\text{SMT}+\partial_{10}\text{SIS}+\partial_{11}\text{GRP}+\partial_{12}\text{TIP}+\partial_{13}\text{HSD}+\varepsilon$$

where *W* represents the information connection matrix, and *PGR*, *UPD*, *CPM*, *ECU*, *HEC*, *ITW*, *RDO*, *RTS*, *SMT*, *SIS*, *GRP*, *TIP*, and *HSD* are the difference matrices formed by each variable. The $\partial_{1},\partial_{2},...\partial_{13}$ represent influence coefficients of this model, and the ε represents the random coefficient.

### 3.2. Data Sources

The BI is a measurement indicator from Baidu’s analysis platform for behavior data of huge Internet users, which represents how frequently specific keywords are entered on Internet search pages [11,12]. The BI originates from the Baidu Index platform http://index.baidu.com (accessed on 22 November 2021), which includes search index and media index. The media index calculates the volume of keywords, which is reported by the Internet media. Furthermore, media index only shows the hotspots reported by the media, which does not fully show the characteristics of Internet searches. Hence, the media index is not very representative. However, the search index calculates the search volume of keyword on the Baidu platform https://www.baidu.com/ (accessed on 22 November 2021). The value of the search index measures the public demands and interests at different times and in different regions, which more authentically represents public search attention on the Internet. Therefore, in this study, the BI refers to the search index.

Particularly, the BI can reflect the public search attention. When the public is interested in a certain city, they will use the name of the city as a keyword searching on the Internet; the BI calculated by this process represents the public search attention to this city. Therefore, we use the correlation between BI and public search attention to characterize the information flow between cities. For example, the BI that is generated by the public in Xi’an of China using “Beijing” as a keyword to search on the Internet represents the information flow from Xi’an of China to Beijing of China.

## 4. Empirical Research

### 4.1. Research Area and Data

**Figure 4:**
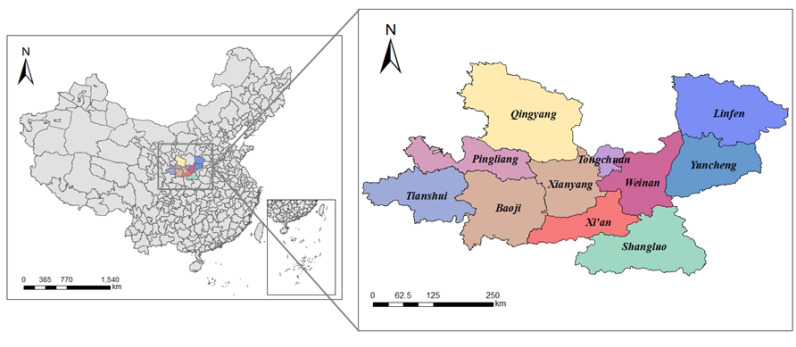
The research area.

The Guanzhong Plain Urban Agglomeration (GPUA) was approved by the State Council of China in 2018, which is the last urban agglomeration. Furthermore, the Development Plan for GPUA issued by the National Development and Reform Commission in China in 2018 delineates 11 prefecture-level cities: Xi’an, Baoji, Xianyang, Tongchuan, Weinan, and Shangluo in Shaanxi Province; Yuncheng and Linfen in Shanxi Province; and Tianshui, Pingliang, and Qingyang in Gansu Province. However, GPUA is located in the northwest of China, where the economic foundation is weak, and the urban development is uneven. Particularly, the State Council of China stated that it is necessary to be concerned about the spatial network structure of GPUA for promoting the socioeconomic development of GPUA. Therefore, analyzing the evolution and driving factors of the network is of great significance for promoting the coordinated cooperation and sustainable development of GPUA. Figure 4 and Table 6 describe the research area.

Based on the above theoretical basis, we collected the data for 11 cities (i.e., cities’ names in GPUA as the specific keywords) in Baidu Index platform from 2014 to 2020. Then, we used the 12-month average of the BI to represent the current year’s information flow among cities for ensuring accuracy and comparability of data. Other socioeconomic statistical indicators used in this study were collected from the “China City Statistical Yearbook”, “Shaanxi Province Statistical Yearbook”, “Shanxi Province Statistical Yearbook”, and “Gansu Province Statistical Yearbook”, such as Population Growth Rate, Urban Population Density, and Annual Mean Concentration of PM2.5.

### 4.2. Spatiotemporal Evolutionary Characteristics of IFN in GPUA

#### 4.2.1. The Distribution Characteristics of the Information Flow

Equations (5) and (6) were respectively used to calculate the information flow and *C_v_* of the whole network in the GPUA from 2014 to 2020. Table 7 shows the results of the trends.

**Figure 5:**
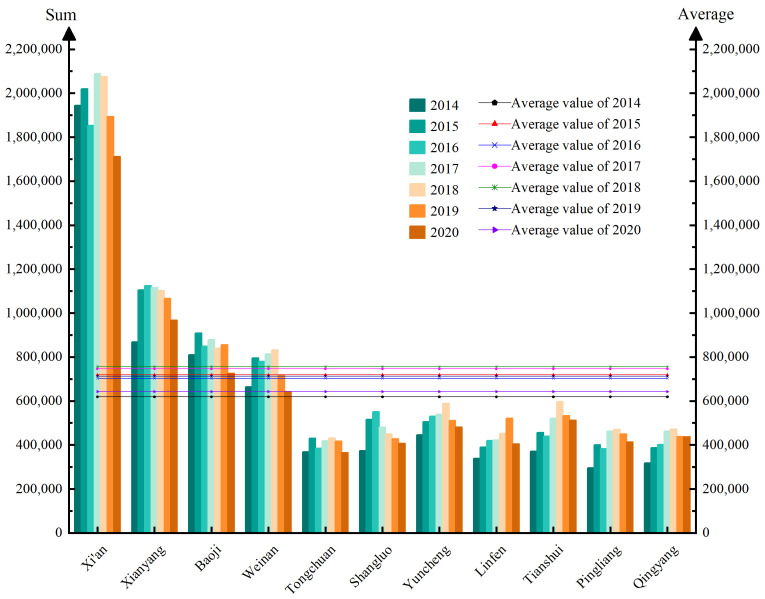
The distribution of information flow of each urban node in GPUA (2014–2020).

The whole information flow in GPUA showed a trend of increasing fluctuations in the early study period and gradually decreasing in the later study period. However, we found that *C_v_* changed from 0.78 to 0.62; the value remained less than 1. This indicated that although the whole information flow in urban agglomerations gradually decreased, the degree of dispersion of information flow decreased year by year. The reason for this phenomenon may be that during 2014–2018, with the economic development of GPUA, attention and interest of residents increased in GPUA, so the information flow in the urban agglomeration area also increased. However, due to the impact of the national economic downturn and the COVID-19 pandemic during 2019–2020, residents of GPUA had to stop working and isolate at home, resulting in activity of people’s production activities declining. Therefore, the information flow continued to decline. The whole information flow in GPUA showed a downward trend under the constraints of the external environment, but the degree of agglomeration of information flow in the urban agglomeration increased for the measures such as industrial cooperation and joint construction. Then, we further analyzed the characteristics of each urban node by measuring the sum and average value of information flow from 2014–2020. Figure 5 shows the results.

The results of Figure 5 obviously show that information flow of each urban node in GPUA also showed a trend of fluctuating upward and then downward. Particularly, the information flow of Xi’an, Xianyang, Baoji, and Weinan all exceeded the average value during the study period, which contributed a lot to the whole information flow in the urban agglomeration. The study period was divided into three important time points for analysis.

(1) In 2014–2015, China continued to promote the development of telecommunications services and Internet services. Due to the government’s policy support, the Internet infrastructure of GPUA developed rapidly, and then the frequency Internet searches by residents increased. Therefore, this stage generated much information flow at a faster rate.
(2) During 2016–2017, except for the decline in information flow in Xianyang and Shangluo, the information flow in other cities showed an increasing trend. Due to the loss of some permanent residents in Xianyang, the resident activation in this area decreased, so the intensity of information flow also decreased. Meanwhile, Shangluo was indirectly affected by the poor economic condition and economic downturns, causing a decrease of information flow. However, with the increase of economic foundation in other cities, urban residents paid more attention to cities with strong economic foundations, promoting the information flow to continue to grow.
(3) From 2018 to 2020, the information flow of each urban node continued to decline during this period, while Baoji and Linfen’s information flow increased and then declined. In 2018, an important event that the Chinese government approved the establishment of GPUA drove the increase of public attention. Therefore, the information flow of GPUA all increased. However, a sudden drop of information flow occurred in 2019–2020 due to poor economic efficiency and the outbreak of COVID-19. During the COVID-19 pandemic, an important source of information flow was “crowd sourcing”, which includes crowd sharing and crowdsourcing logistics. Some urban residents posted the regional epidemic situation with the form of crowd sharing on the Internet, which provided information supports for epidemic monitoring [56,57,58]. Meanwhile, urban residents were quarantined at home paid much attention to the epidemic situation in various regions based on crowd sharing, which generated a large amount of information flow. Furthermore, due to restrictions on urban production activities and work stoppages, some urban residents match transportation demand information released on the Internet to carry out part-time transport distribution activities [59,60]. Particularly, by the way of “crowdsourcing logistics”, urban residents focus on the transportation demand information released within the urban agglomeration to alleviate the negative impact of COVID-19 and obtain some financial supports. The form of “crowd sourcing” reduced the negative effects of COVID-19 to a certain extent, promoting the sharing and exchange of information flow in special period. Meanwhile, “crowd sourcing” also gained some public attention and generated much information flow. However, the negative impacts of COVID-19 on information flow were much higher than the positive impacts. Therefore, the final information flow of cities was relatively reduced.

#### 4.2.2. The Hierarchical Structure of IFN in GPUA

Based on the research method defined in Section 3.1.1, we constructed the IFN and calculated the information flow of GPUA from 2014 to 2020. To identify the hierarchical structure of the IFN, we also divided the information flow of GPUA from 2014 to 2020 into four levels. Table 8 shows the level of hierarchical structure.

**Figure 6:**
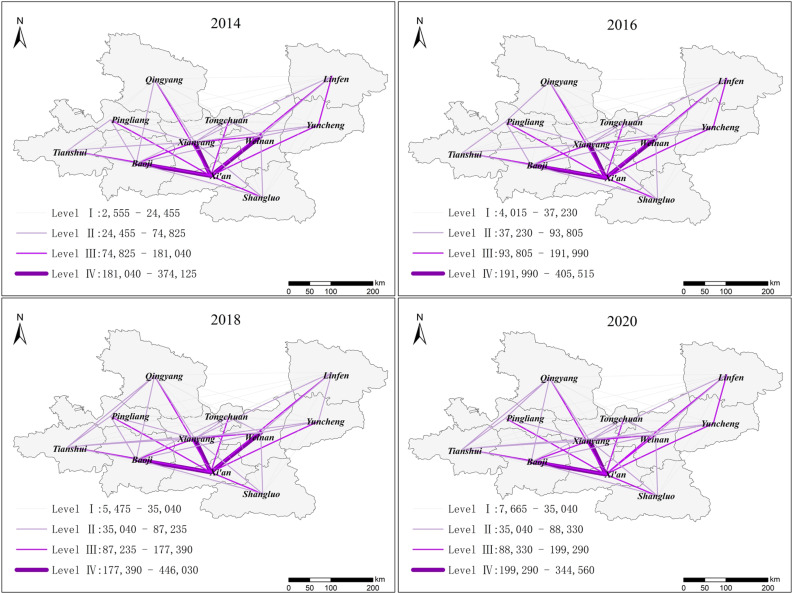
The hierarchical structure of IFN (2014–2020).

Then, we imported the information flow of 11 urban nodes from 2014–2020 into ArcGIS for visualization, observing the evolutionary characteristics of the hierarchical structure. Figure 6 shows the results of hierarchical structure over time.

The results showed that the information flow of central region in GPUA was closer, while the information flow of western and eastern regions were relatively sparse. The characteristics of information flow in GPUA showed a trend of “high in the middle and low on both sides”. This showed that the residents’ search attention hotspots were related to the level of urban economic development. The central region is located in the core area of GPUA. Furthermore, the abundant resources and active population of the central region have advantages, which generated much information flow. However, residents pay less attention to eastern and western regions and generate less information flow for remote location, low socioeconomic status, and low urban influence of these regions.

Xi’an, Xianyang, Baoji, and Weinan firstly formed the core network structure, which was described as a backbone structure of “one core node and three main nodes”. As a central city and historical capital in western China, Xi’an occupied an important position and far surpassed other cities in terms of international influence and social resources. Therefore, Xi’an was the core node in the IFN. The strategy of “Xi’an–Xianyang” integration adopted by the Chinese government has promoted closer exchanges between Xianyang and Xi’an. Furthermore, as a heavy industrial city and the neighbors of Xi’an in the region, Baoji has unique economic advantages; Meanwhile, Weinan is close to Xi’an with a high level of urban construction. Therefore, Xianyang, Baoji, and Weinan as the three main nodes generated high-intensity information flow with Xi’an, which formed a stable network core structure in GPUA.

### 4.3. Analysis of the Node Hierarchy of IFN in GPUA

#### 4.3.1. The Structural Holes Analysis

**Figure 7:**
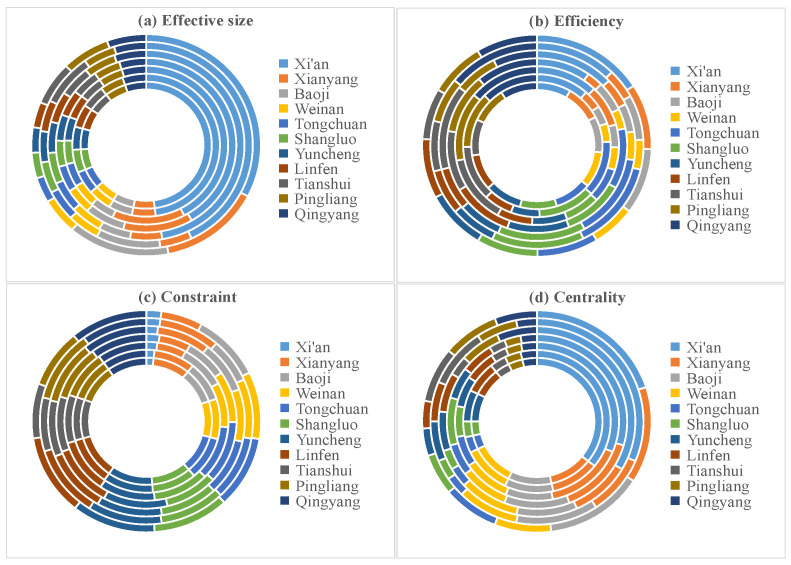
The indicators of structural holes theory in GPUA (2014–2020).

Based on the structural holes method defined in Section 3.1.2, we used the structural holes algorithm of Ucinet6.0 to measure the control and influence of network nodes in the IFN of GPUA. Figure 7 shows the calculation results. The inside circle represents 2014; the outside circle represents 2020, with the other years falling between in sequence.

Figure 7 shows that Xi’an, Xianyang, and Baoji attain larger effective size, while Yuncheng, Linfen, Tianshui, Pingliang, and Qingyang attain low effective size. This is mainly because the effective size represents the control and influence of information flow, which will be affected by the basic attributes of the urban nodes (such as economy, resources, social status, etc.). As the core nodes in the network structure, Xi’an, Xianyang, and Baoji have higher control force and status of information flow than other urban nodes. However, the urban nodes such as Yuncheng, Linfen, Tianshui, Pingliang, and Qingyang are lower in the ranking of Chinese urban strength, with restricted resource conditions. Hence, the control force and status of information flow about Yuncheng, Linfen, Tianshui, Pingliang, and Qingyang were the weakest.

However, Yuncheng, Linfen, Tianshui, Pingliang, and Qingyang showed higher efficiency, because these cities only generated information flow with a small number of neighboring cities with strong resource conversion capabilities. However, Tongchuan and Shangluo were subject to the highest constraint of information flow for their weakest economic foundation in Shaanxi Province. Furthermore, Tongchuan and Shangluo had a small number of neighboring cities without economic and resource assistance, exhibiting more restrictive and dependent conditions. The centrality represents the urban nodes’ core degree of the information flow in the network structure. The central trend decreased from the center toward two extreme points. The centrality of urban nodes in the central region was higher, while the centrality of urban nodes in western and eastern regions was lower. The result of centrality showed a great correlation with geographical location.

#### 4.3.2. Dominance and Control Analysis

**Figure 8:**
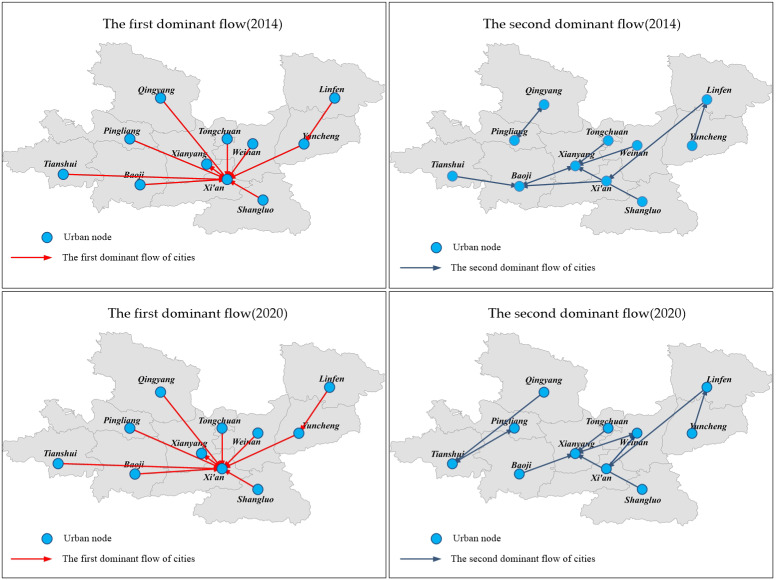
The first dominant flow and second dominant flow of GPUA.

We used the dominance flow analysis to explore the node hierarchy. Figure 8 shows the results.

The results showed that Xi’an received more first dominant flows, connecting with 10 other urban nodes. Xianyang, Baoji, Weinan, Tongchuan, Shangluo, Yuncheng, Linfen, Tianshui, Pingliang, and Qingyang were all affected the most by Xi’an. Furthermore, Xi’an received more than half of the first dominant flows, indicating that Xi’an was the dominant city with the strongest control in the urban agglomeration. From the perspective of the second dominant flow, the trend of second dominant flow was more scattered than first dominant flow during 2014–2020. Xi’an, Xianyang, Baoji, Weinan, Linfen, Tianshui, and Pingliang were all part of the second dominant flow. According to the results, Xi’an was the dominant city, and the other 10 cities were subordinate cities.

These outcomes indicated that the Xi’an was the core node of IFN in GPUA, and the node hierarchy of other cities was very different from Xi’an. The reason was that the node hierarchy was related to the actual urban planning and socioeconomic status. As the only key city in the economic belt of northwestern China, Xi’an has obvious geographical advantages, abundant resources, and a solid foundation for industrial development. The other cities in GPUA are quite different from Xi’an in social and economic ability. Meanwhile, resources of the other 10 cities such as transportation, industry, and technology are heavily dependent on Xi’an. Therefore, the first dominant flows of these cities were all to the Xi’an. However, the second dominant flow was more likely to be generated between adjacent cities, indicating that the information flow between adjacent cities had a larger scale, which also showed the characteristics of geographic proximity. The geographical space influenced the formation of IFN, promoting the information flow among the administrative regions more closely.

### 4.4. Cohesive Subgroup Analysis

**Figure 9:**
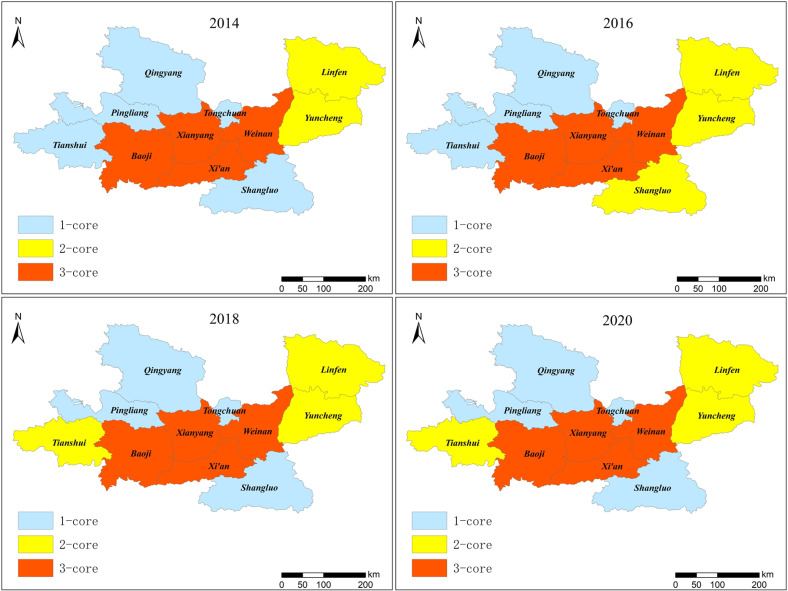
The subgroups of GPUA (2014–2020).

We applied the *k*-core algorithm to analyze the network structure, using Ucinet 6.0 to perform a cohesive subgroup analysis on IFN in GPUA from 2014 to 2020. The *k*-core algorithm divided the urban nodes of IFN in the GPUA into three subgroup levels, with *k* values of 1, 2, and 3. Figure 9 shows the results.

Figure 9 shows that the internal spatial agglomeration of information flow in GPUA strengthened and became closer from 2014 to 2020. At the beginning of the study period, the four cities of Xi’an, Xianyang, Baoji, and Weinan formed 3-core subgroups; the two cities of Linfen and Yuncheng formed 2-core subgroups; and the other five cities of Tongchuan, Shangluo, Tianshui, Pingliang, and Qingyang formed 1-core subgroups. The structure of cohesive subgroups reflects the aggregation and reciprocity of information flow in the urban interaction. The agglomeration degree of cohesive subgroups was still spreading from the central region to the east and west regions. Furthermore, the formation of cohesive subgroups showed an obvious geographic characteristic, which was constrained by provincial and administrative boundaries, resulting in closer ties among neighboring cities.

At the final study period, there was a relative increase in the number of cities in 2-core subgroups and a relative decrease in the number of cities in 1-core subgroups. The reason was that the three provinces of Shaanxi, Shanxi, and Gansu continued to cooperate in industry to jointly build major infrastructure in GPUA, which had promoted the aggregation of cohesive subgroups. Meanwhile, to adjust the industrial structure, Tianshui transformed and upgraded their traditional industries, which significantly improved the economic level and intensity of information flow, leading to a significant increase in the cohesion of subgroups. Shangluo also promoted the construction of a new urbanization and development of tourism economy, which increased the information flow. Therefore, policy assistance, industrial transformation, infrastructure, and other measures are the keys to increase the interaction and cohesion among urban nodes in GPUA.

### 4.5. The Sustainable driving Factors of IFN in the GPUA

#### 4.5.1. The QAP Correlation Analysis

For this study, we first selected 5000 random permutations for replacement in the matrices based on the different networks involved in the sustainable driving factors of IFN. Then, we conducted the correlation analysis of IFN and the driving factors. Table 9 shows the results.

The correlation coefficients in Table 8 indicate the degree of correlation between the dependent variable (W) and independent variable (PGR, UPD, CPM, ECU, HEC, ITW, RDO, RTS, SMT, SIS, GRP, TIP, HSD) matrices. The maximum and minimum values reflect the actual correlation coefficient values of the 5000 random permutations. Values of *p* ≥ 0 and *p* ≤ 0 indicate that the observed correlation coefficients in the 5000 random permutations were greater than or less than the probability of the actual correlation coefficient.

Table 9 shows that the driving factors are all positively correlated to the IFN of GPUA. The PGR, UPD, ECU, SMT, SIS, GRP, and TIP are at a 1% significance level; the CPM, HEC, ITW, RDO, RTS, and HSD are at a 5% significance level. The significance level of correlation coefficients passed the test, indicating that the variables of public ecology, resource investment, information infrastructure, and economic foundation were all strongly correlated to IFN. The results also showed that the four driving factors, public ecology, resource investment, information infrastructure, and economic foundation, were strongly related to information flow, which had a greater impact on information flow. Particularly, due to urban ecological problems (global warming, traffic producers, heavy smog pollution, etc.), urban residents are worried about the ecological situation of the living environment and paying more attention to the public ecology. Public ecology became a key topic of public search, which was positively related to information flow. The indicators of resource input were positively related to information flow, which was related to the facility support of information flow, such as human resources, energy consumption, and technology. Information infrastructure was the guarantee for residents to use the Internet and provide support for the public online search behavior, which was positively related to the flow of information. The economic foundation reflected the living standards of residents and was positively correlated with information flow.

#### 4.5.2. The QAP Regression Analysis

To further explore the mechanism involved in the formation of IFN, we conducted a QAP regression analysis based on the QAP correlation analysis. The QAP regression analysis relied on the relationship matrix through 10,000 random permutations and repeated calculations. Then, we studied the relationship among multiple independent variable matrices and the dependent variable matrix. Table 10 shows the results.

Table 10 shows that the adjusted *R*^2^ was 0.693, which was significant at the 1% level. The *R*^2^ indicated that the driving factors explained 69.3% of the IFN in GPUA, which were credible results. The CPM, RDO, GRP, and TIP variables were significant at a 1% level, indicating these variables had important impacts on the IFN in GPUA. The PGR, UPD, ECU, SMT, and SIS variables were significant at a 5% level, indicating they also impacted the IFN in GPUA. The variables of public ecology and information infrastructure all pass the significance test, indicating public ecology and information infrastructure provided strong explanations for the improvement of information flow. Common ecological problems in the urban living environment, including air pollution caused by transport generators and industrial production, affected the ecological environment of urban residents. This caused urban residents to pay much attention to ecology and generate much information flow. In fact, with the measures of ecological civilization promoted by the government, urban residents obviously paid a lot of attention to the public ecological construction of the living environment. Meanwhile, the support of information infrastructure provided a solid foundation for the dissemination and interaction of information flow.

Furthermore, the PGR, UPD, CPM, ECU, RDO, SMT, SIS, GRP, and TIP variables played an important role in IFN; particularly, the SMT, RDO, GRP, and UPD variables also showed large regression coefficients. This showed that these factors with strong effects attracted the residents’ main attention, which also was the key factor that residents care about in urban construction. However, other variables, such as HEC, ITW, and HSD, did not pass the significance test. This does not mean that these variables did not affect IFN; the reason of this result is that gradual improvements in the urban economic foundation and resource inputs gradually weakened their decisive role in IFN. Instead, public ecology and information infrastructure became a more important factor.

## 5. Discussion

In the information age, people’s information flow becomes more intensive, and the interaction of intercity network has also undergone major changes. However, there is currently a lack of exploration of the intercity network from the perspective of information flow. We have filled this gap and used the public search attention to characterize the intercity information flow, proposing a research framework for exploring the evolution of network and driving factors based on the BI. It is worth noting that the research framework of this study is extensible and can be applied to other cities, which can be used to further explore the movement of internal and external information flow in cities. This study contributed to further application of big data generated by people on the Internet, which also enriched the research on the characteristics of intercity network. The specific research findings are summarized in Table 11.

According to the research results in Table 11, we have the following interesting findings:

(1) Comparing with urban socioeconomic statistics, we found that the empirical results of spatiotemporal evolution and node hierarchy of IFN are consistent with the actual social and economic conditions of cities in GPUA. For example, Xi’an is a central city in northwestern China, which is also the core node in IFN; Qingyang and Pingliang are the weakest cities in GPUA and are still the marginalized node in IFN. This shows that urban socioeconomic conditions and information flow are positively correlated. We choose the three indicators of economic foundation driving factors as representatives of socioeconomic statistics to examine the correlation, which is verified in Section 4.5.1. The results of correlation analysis are shown in Table 12.

Table 12 shows that urban nodes with high socioeconomic status will generate a large-scale information flow in IFN and in a higher hierarchical structure, which is consistent with the finding drawn by Li [39].The reason is that public search attention of urban residents is affected by the socioeconomic conditions of the city. The city with strong socioeconomic conditions will be accompanied by a high urban visibility. Furthermore, urban residents will show a higher degree of attention to the city and generate more information flow, which impacts on the evolution and node hierarchy in the IFN. This also shows that the characteristics and attributes of the city will affect the social behavior of human beings, and in turn, the social behavior of humans affects the characteristics and attributes of the city.

(2) In the information age, the evolution of the intercity network is still limited by geographic distance. The research results of cohesive subgroups show that the agglomeration among neighboring cities is stronger, which makes it easier to form agglomerated subgroups with obvious regional spatial characteristics. For example, the urban nodes in the central region of GPUA, such as Xi’an, Xianyang, Baoji, and Weinan, with closer geographical distance and superior location, quickly formed a cohesive subgroup. This feature can be explained in the research of Wu et al. [40]: longer geographic distance will weaken the spread of information flow, while the information flow between neighboring cities has a strong agglomeration. The reason is that due to population migration, convenient transportation, and closer policy cooperation among neighboring cities, the information flow exchanges frequently. In the frequent policy cooperation environment, urban residents will show a higher degree of attention, which will generate more information flow.
(3) Compared with previous research results, the results of this paper show that the indicators of public ecology and information infrastructure have a strong ability to explain information flow [39,40,41]. For example, PGR, UPD, and CPM of public ecology increased by 1%; meanwhile, the information flow increased by 0.202%, 0.358%, and 0.215%, respectively. The RTS, SMT, and SIS of information infrastructure increased by 1%, while information flow increased by 0.131%, 3.244%, and 0.168%, respectively. Therefore, public ecology, resource investment, information infrastructure, and economic foundation are important factors affecting the IFN; public ecology and information infrastructure are especially key factors. This shows that with the continuous development of urban economy, resource investment and economic foundation are no longer the key factors restricting information flow, while public ecology and information infrastructure are the key factors affecting information flow. With the continuous increase of population size and population density, the urban resources have decreased, and environmental pollution has increased. The ecological pollutions caused by transport generators and industrial producers have aroused public attention, generating much information flow. Furthermore, with the rise of the national quality education level, the public self-awareness has strengthened, paying much attention to the public ecology and the livability of the urban environment, including public environment, public services, and public resources. On the other hand, information infrastructure, as a medium for the generation and dissemination of information flow, is the basis for ensuring the flow of information flow in intercity networks. Therefore, to promote coordination and cooperation between cities and sustainable development, the construction of public ecology and information infrastructure is the key.

In this study, we used BI as data support to construct the IFN from the perspective of public search attention, which provides a feasible framework reference for the construction of intercity networks in the information age. Meanwhile, we built sustainable driving factors to explore the influence of the IFN from public ecology, resource investment, information infrastructure, and economic foundation. This provides a theoretical support for the scientific analysis of key factors affecting the urban sustainable development. Meanwhile, the research results reflect the relevance of the IFN to the urban socioeconomic status and geographic characteristics, which further explain the formation mechanism of the intercity network. This also provides a decision-making support for government departments to formulate urban interactive cooperation strategies in the information age. Furthermore, the research results identifying the core nodes and subgroups of the intercity network provide a decision support for government departments to narrow the gap and shortcomings among cities. Finally, the research results conducted by the correlation and regression analysis of sustainable driving factors provide a basis for government departments to adopt targeted and efficient measures in promoting the urban sustainable development.

## 6. Conclusions

In the era of Internet, the evolution of the intercity spatial structure is influenced by the information flow. To measure it, we used BI, a tool that can quantify public attention from an Internet search perspective. Based on the quantification results, we proposed a new framework to explore the evolution of intercity network and driving factors of information flow. The results show that the information flow of GPUA increases in fluctuation and then gradually decreases; the network hierarchical structure and node hierarchy are related to the actual urban influence; the formation of cohesive subgroups depends closely on the geographical location; and the public ecology and information infrastructure has a strong impact on the sustainability of IFN. Our policy recommendations are listed as follows: 

First, the central cities with strong controlling force should play a radiating role by establishing urban industrial clusters to facilitate information flow with neighboring cities. Industrial development provides a material basis for the development of IFN in the urban agglomeration. For example, economically developed areas such as Xi’an, Xianyang, Baoji, and Weinan should jointly establish urban industrial clusters and adjust the industrial structure, promoting the growth of information flow.

Second, the information flow in urban agglomerations shows the obvious characteristics of spatial clustering. Furthermore, marginal cities produce less information flow, while central cities produce more information flow. Therefore, the government should consider the impacts of economic development, traffic conditions, and the geographic location on IFN. All the new approach should focus on breaking geographical boundaries and promoting politicoeconomic cooperation.

Finally, public ecology, resource investment, information infrastructure, and economic foundation are important factors affecting sustainable development of the IFN. In particular, the role of public ecology and information infrastructure in affecting the sustainable development of IFN gradually strengthened. Therefore, when the government invests in resource and economic foundation, it should further improve public ecology and information infrastructure.

From the perspective of public search attention, we explored the evolution and the sustainable driving factors of IFN and provided theoretical and practical support for the coordinated development of intercity networks in the information age. However, this study only used BI as a measure of public search attention to construct an IFN and explore the evolution of intercity networks. In the future, we will use multiple Internet behavior data to expand the connotation of the IFN to comprehensively reflect the characteristics of the IFN. This study focused on exploring the evolution and driving factors of IFN. Meanwhile, we will expand the study of intercity networks constructed by other material element flows (traffic flow, people flow, logistics, etc.) and compare the research results of IFN with those of other element flow networks. This will further explore the differences of intercity network evolution from different perspectives and provide more practical supports for intercity networks.

## Figures and Tables

**Table 1:** Comparison of Baidu Index and Google Trend.

	Baidu Index	Google Trend
Source	Baidu search engine—the world’s largest Chinese engine	Google search engine—the world’s largest search engine
Indicators	Weighted sum of keyword search frequency in Baidu	Relative propensity of users to search for a topic on Google
Numerical Range	0~infinity	0~100
Features	Real-time update, convenient, and fast	Fast update speed and wide-spread
Precision	More accurate identification of retrieval content	Relatively accurate retrieval of content
Application Scope	Keyword search trends, insight into interests and needs	Monitor and predict the regional popularity of hot topics

**Table 2:** The specific meaning of typical structural holes indicators.

Indicators	Specific Meaning
Effective size	Measure node’s degree of occupying resources and whole impact in structural holes.
Efficiency	Measure the node’s conversion efficiency on other nodes in the network.
Constraint	Measure the node’s dependence degree on other nodes.
Hierarchy	Measure agglomeration of the node subject to the constraints of other nodes
Centrality	Measure the core level of nodes in the network structure.

**Table 3:** The calculated process of dominance flow analysis.

Process	Dominance Flow Analysis to Identify the Hierarchy of Urban Nodes
1:	Calculate the dominant flows from node 1 to node *n* in sequence (*0* < *j* <*n)*
2:	For node 1:
3:	sort the information flow generated by node 1: *r*_11_, *r*_12_, *r*_13_*...*, *r*_1*n*_
4:	first dominant flow of node 1:
5:	if the *r*_1*j*_ *== Max (r*_11_, *r*_12_, *r*_13_*...*, *r*_1*n*_*)*
6:	the *FDM*_1*j*_ = *r*_1*j*_
7:	the *FDM*_1*j*_ represents the first dominant flow sending from node 1 to node *j*
8:	indicating node *j* receives a first dominant flow
9:	second dominant flow of node 1:
10:	delete *r*_1*j*_ from *r*_11_, *r*_12_, *r*_13_*...*, *r*_1*n*_
11:	if the *r*_1*j’*_ *== Max (r*_11_, *r*_12_, *r*_13_*...*, *r*_1*n*_*)*
12:	the *SDM*_1*j ’*_*= r*_1*j’*_
13:	the *SDM*_1*j’*_ represents the second dominant flow sending from node 1 to node *j′*
14:	indicating node *j′* receives a second dominant flow
15:	Repeat steps 2~14 to calculate each urban node
16:	Then count the first dominant flow and second dominant flow received by each node
17:	Identify the dominant node, subdominant node, and subordinate node

**Table 4:** The affiliation of urban nodes.

Urban Affiliation	Definition
Dominant node	receives at least 50% the first dominant flow
Subdominant node	receives at least 50% the second dominant flow
Subordinate node	did not receive any dominant flow

**Table 5:** The sustainable driving factors of IFN.

Driving Factors	Variable Name	Symbol	Unit
Public ecology	Population Growth Rate	PGR	%
	Urban Population Density	UPD	households/sq.km
	Annual Mean Concentration of PM2.5	CPM	ug/m^3^
Resource investment	Energy Consumption per unit of GDP	ECU	10,000 households
	Household Electricity Consumption	HEC	10,000 yuan
	Information Technology Workers	ITW	%
	R&D Internal Outlay	RDO	10,000 KWh
Information infrastructure	Revenue from Telecommunication Services	RTS	10,000 yuan
	Number of Mobile Telephone Subscribers	SMT	10,000 households
	Number of Internet Services Subscribers	SIS	10,000 households
Economic foundation	Per Capita GDP	GRP	yuan
	Tertiary Industry as Percentage of GDP	TIP	%
	Household Saving Deposits	HSD	yuan

**Table 6:** The regions of the Guanzhong Plain Urban Agglomeration (GPUA).

**City**	Qingyang Pingliang Tianshui	Tongchuan Xianyang Baoji	Weinan Xi’an Shangluo	Linfen Yuncheng
**Region**	Western region	Central region		Eastern region

**Table 7:** The information flow and *C_v_* of the GPUA (2014–2020).

	2014	2015	2016	2017	2018	2019	2020
Information flow	6,802,140	7,916,120	7,718,290	8,207,390	8,311,050	7,832,170	7,070,050
*C_v_*	0.78	0.69	0.64	0.67	0.65	0.62	0.62

**Table 8:** The hierarchical level of the information flow.

Level	Hierarchy	Indication
Level I	Weak information flow	Two cities were not very closely connected
Level II	Lower levels of information flow	Two cities were closely connected
Level III	Medium information flow	Two cities were very closely connected
Level IV	Strong information flow	Two cities maintain the largest information flow

**Table 9:** The correlation analysis of GPUA.

Variable	Correlation Coefficient	Significance Level	Standard Deviation	Minimum	Maximum	Prop ≥ 0	Prop ≤ 0
PGR	0.306 ***	0.005	0.099	−0.221	0.366	0.005	0.996
UPD	0.471 ***	0.008	0.21	−0.393	0.531	0.008	0.992
CPM	0.747 **	0.011	0.253	−0.253	0.767	0.011	0.989
ECU	0.242 ***	0.006	0.071	−0.236	0.263	0.994	0.006
HEC	0.201 **	0.04	0.115	−0.141	0.233	0.04	0.96
ITW	0.618 **	0.023	0.268	−0.2	0.76	0.051	0.949
RDO	0.680 **	0.040	0.246	−0.314	0.74	0.041	0.959
RTS	0.752 **	0.046	0.22	−0.317	0.688	0.008	0.992
SMT	0.631 ***	0.006	0.255	−0.301	0.765	0.051	0.95
SIS	0.736 ***	0.007	0.253	−0.302	0.769	0.008	0.992
GRP	0.733 ***	0.002	0.246	−0.268	0.76	0.002	0.998
TIP	0.522 ***	0.002	0.182	−0.417	0.57	0.002	0.998
HSD	0.756 **	0.015	0.263	−0.224	0.787	0.015	0.987

** indicates significant at the 5% confidence level; *** indicates significant at the 1% confidence level.

**Table 10:** The regression analysis of GPUA.

Variable	Nonstandardized Coefficient	Standardized Coefficient	Significance	Probability 1	Probability 2
PGR	0.230	0.202 **	0.020	0.981	0.02
UPD	0.001	0.358 **	0.038	0.038	0.96
CPM	0.195	0.215 ***	0.009	0.992	0.01
ECU	0.173	0.189 **	0.029	0.971	0.03
HEC	0.000	1.295	0.343	0.658	0.34
ITW	0.359	0.305	0.291	0.291	0.71
RDO	0.460	0.451 ***	0.002	0.998	0.00
RTS	0.119	0.131 **	0.075	0.925	0.08
SMT	0.122	3.244 **	0.016	0.985	0.02
SIS	0.153	0.168 **	0.029	0.972	0.03
GRP	0.405	0.444 ***	0.001	0.001	1.00
TIP	0.362	0.229 ***	0.008	0.993	0.01
HSD	0.360	0.317	0.102	0.102	0.90

** indicates significant at the 5% confidence level; *** indicates significant at the 1% confidence level.

**Table 11:** The research findings for this study.

Dimension	Measure	Finding
Whole network	Evolution characteristics	The information flow in GPUA fluctuates up and then down, but the degree of clustering continues to grow.
	Hierarchical structure	Information flow showed a trend of “high in the middle and low on both sides”, while node hierarchy showed a “1 + 3 + 7” hierarchical distribution.
Node hierarchy	Structural holes	Effective size, efficiency, constraints, and centrality are related to the core degree of the urban node.
	Dominance flow	Xi’an was the only dominant city, while other 10 cities were subordinate cities.
Cohesive subgroup	The *k*-core algorithm	The formation of cohesive subgroups was constrained by geographical distance.
Driving factor	QAP analysis	The public ecology and information infrastructure became the important factor affecting IFN.

**Table 12:** The correlation analysis between information flow and socioeconomic statistics.

	Information Flow	GRP	TIP	HSD
Correlation Coefficient	1	0.733 ***	0.522 ***	0.756 **
Significance Level	-	0.002	0.002	0.015

** indicates significant at the 5% confidence level; *** indicates significant at the 1% confidence level.

## Data Availability

The data used to support the findings of this study are available from the corresponding author upon request.

## Abbreviations

The following abbreviations are used in this manuscript:

BI	Baidu Index
GPUA	Guanzhong Plain Urban Agglomerations
IFN	Information Flow Network
QAP	Quadratic Assignment Procedure
